# Ether in Labor

**Published:** 1874-11

**Authors:** 


THE USE OF ETHER DURING LABOR.—With regard to the action of ether, Dr. Smith remarked that many cases present a condition of spasmodic contraction of the neck of the uterus, in which anesthetics have an admirable effect. In these cases labor goes on with violent contraction, and the os uteri will not relax when the head presses upon it. Here ether will be of service by its property of reducing relaxation.

In other cases he thought that ether retards labor by enfeebling the power of the patient. In *multiparæ,* where the os is in a yielding condition, there is no reason to expect delay from that source; hence ether retards labor by impairing the voluntary contractions which are so useful. The patient cannot bear down, because consciousness is impaired and volition is absent. If the patient insists, we may use ether as a placebo, only upon condition that she will bear down.

The prolonged use of ether will impair the vitality of the fetus. He had rarely seen a case in which the use of ether was prolonged in which the child did not require some effort to revive it.—*The Obstetrical Journal.*

ALWAYS employ a saturated infusion of coffee in opium poisoning. It is always at hand, and can be used while other remedies are being prepared.—*Medical and Surgical Reporter.*

The birth-rate in the United Kingdom in the second quarter of 1874 was 35.7 per 1,000, and the death-rate 20.8. The marriage rate in the first quarter was 14.4 per 1,000.

